# The New Synthetic H_2_S-Releasing SDSS Protects MC3T3-E1 Osteoblasts against H_2_O_2_-Induced Apoptosis by Suppressing Oxidative Stress, Inhibiting MAPKs, and Activating the PI3K/Akt Pathway

**DOI:** 10.3389/fphar.2017.00007

**Published:** 2017-01-20

**Authors:** Xiaofei Yan, Haixia Wu, Zhiyuan Wu, Fei Hua, Dong Liang, Hong Sun, Yong Yang, Dejian Huang, Jin-Song Bian

**Affiliations:** ^1^Department of Biochemistry and Molecular Biology, Medical College of Xi’an Jiaotong UniversityXi’an, China; ^2^Department of Pharmacology, Yong Loo Lin School of Medicine, National University of SingaporeSingapore, Singapore; ^3^Department of Food Science, Faculty of Science, National University of SingaporeSingapore, Singapore; ^4^Department of Physiology, Xuzhou Medical UniversityXuzhou, China; ^5^State Key Laboratory of Natural Medicines, Jiangsu Key Laboratory of Drug Discovery for Metabolic Disease, Center for New Drug Safety Evaluation and Research, China Pharmaceutical UniversityNanjing, China

**Keywords:** reactive oxygen species, hydrogen sulfide donating drugs, osteoblast, MAPK signaling, PI3K/AKT

## Abstract

Reactive oxygen species (ROS) are important in osteoporosis development. Oxidative stress induces apoptosis of osteoblasts and arrest of their differentiation. Both Danshensu (DSS) and hydrogen sulfide (H_2_S) produce significant antioxidant effect in various systems. In this study, we synthesized SDSS, a novel H_2_S-releasing compound derived from DSS, and studied its antioxidant effect in an H_2_O_2_-induced MC3T3-E1 osteoblastic cell injury model. We first characterized the H_2_S releasing property of SDSS in both *in vivo* and *in vitro* models. HPLC chromatogram showed that intravenous injection of SDSS in adult rats released ADT-OH, a well proved H_2_S sustained-release moiety, within several minutes in the rat plasma. Using an H_2_S selective fluorescent probe, we further confirmed that SDSS released H_2_S in MC3T3-E1 osteoblastic cells. Biological studies revealed that SDSS had no significant toxic effect but produced protective effects against H_2_O_2_-induced MC3T3-E1 cell apoptosis. SDSS also reversed the arrest of cell differentiation caused by H_2_O_2_ treatment. This was caused by the stimulatory effect of SDSS on bone sialoprotein, runt-related transcription factor 2, collagen expression, alkaline phosphatase activity, and bone nodule formation. Further studies revealed that SDSS reversed the reduced superoxide dismutase activity and glutathione content, and the increased ROS production in H_2_O_2_ treated cells. In addition, SDSS significantly attenuated H_2_O_2_-induced activation of p38-, ERK1/2-, and JNK-MAPKs. SDSS also stimulated phosphatidylinositol 3-kinase/Akt signaling pathway. Blockade of this pathway attenuated the cytoprotective effect of SDSS. In conclusion, SDSS protects MC3T3-E1 cells against H_2_O_2_-induced apoptosis by suppressing oxidative stress, inhibiting MAPKs, and activating the phosphatidylinositol 3-kinase/Akt pathway.

## Introduction

Osteoporosis is a disease in which bones become fragile, leading to increased bone fracture risk. At present, 200 million people are estimated to suffer from osteoporosis worldwide (Cooper, 1999). Although the pathogenesis of osteoporosis is still not fully understood, recent findings support that reactive oxygen species (ROS) are important in the development of osteoporosis (Basu et al., 2001; Isomura et al., 2004; Manolagas, 2010). High levels of lipid peroxidation, along with low activities of antioxidant enzymes, were reported in the blood of postmenopausal osteoporotic women and in the femora of ovariectomized rats (Maggio et al., 2003; Muthusami et al., 2005).

In all cases of osteoporosis, there is an imbalance between bone resorption and bone formation. In bones, osteoblasts are responsible for bone formation and osteoclasts are associated with bone degradation. Osteoporosis can occur when bone formation decreases and bone resorption increases or remains unchanged, leading to a net bone loss (Isomura et al., 2004). ROS accelerate apoptosis of osteoblasts, inhibit their differentiation, and impair bone formation (Bai et al., 2004; She et al., 2014; Li et al., 2015).

Danshensu [β-(3, 4-dihydroxyphenyl)lactic acid] is a prominent water-soluble compound extracted from Radix *Salviae miltiorrhizae* (known as ‘Danshen’ in Chinese). It has been widely used in clinics in China for the treatment of various microcirculatory disturbance-related diseases (Yu et al., 2012). DSS reduces inflammation and suppresses ROS formation (Li et al., 2012; Lu et al., 2014; Jiang et al., 2015). Apart from these effects, DSS also produces beneficial effects on bone formation. For example, previous studies revealed that DSS not only stimulated osteoblast differentiation and bone matrix formation, but also oriented rMSCs to osteogenesis (Cui et al., 2009; Guo Y. et al., 2014).

Hydrogen sulfide (H_2_S), an endogenous gaseous mediator, is produced by pyridoxal-5′-phosphate-dependent enzymes, including cystathionine γ-lyase (CSE), CBS and 3-MST during the cysteine metabolism (Hu et al., 2011; Liu et al., 2014). Along with nitric oxide (NO) and carbon monoxide (CO), H_2_S plays multiple physiological and pathological functions in various biological systems (Hu et al., 2011; Liu et al., 2014). It exerts protective effects against various stimuli-triggered injuries. For example, H_2_S protects neurons against neurotoxins-induced brain injury (Tay et al., 2010; Lu et al., 2012). H_2_S also decreases the mortality of myocardial cells during ischemia-reperfusion injury or metabolic inhibition (Pan et al., 2006, 2008; Sivarajah et al., 2006; Yong et al., 2008a,b). A recent study from our laboratory revealed that H_2_S protects osteoblastic cells from H_2_O_2_-induced cell injury (Xu et al., 2011). All the aforementioned biological effects are related to the antioxidant effects of H_2_S.

**FIGURE 1:**
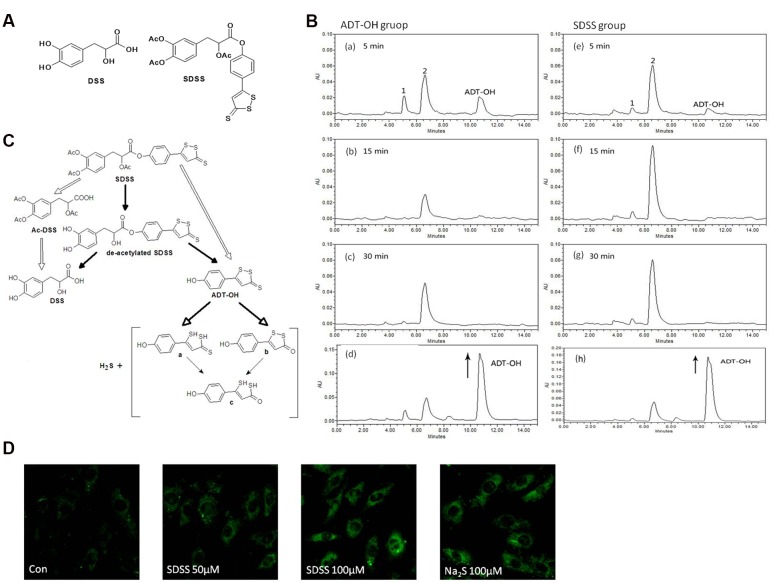
**Characterization of the new H_2_S releasing compound SDSS. (A)** Structural formulas of DSS and SDSS. SDSS is a hybrid of Ac-DSS and ADT-OH. **(B)** HPLC chromatograms of SDSS. Rats were administered with 30 mg/kg ADT-OH (a-c) or SDSS (e–g). Chromatograms were recorded at 330 nm by analyzing plasma 5, 15, and 30 min after injection. For the bottom panels (d and h), samples from ADT-OH (d) and SDSS (h) groups were spiked with additional solution of ADT-OH in ACN (with the arrow) and immediately analyzed by HPLC. The final concentrations of spiking ADT-OH in sample ADT-OH and SDSS were 0.01 mM and 0.02 mM, respectively. **(C)** Proposed diagram of metabolic degradation of SDSS and the release of H_2_S. The metabolic degradation of AFT-OH was proposed by Giustarini et al. (2010a). **(D)** Detection of intracellular H_2_S released from SDSS in MC3T3-E1 cells. Cells were pretreated with probe 8 (20 μM) in DOTAP liposome for 2 h, and then treated with SDSS or Na_2_S for 3 h. Representative fluorescent images were taken using a confocal microscopy. Ex = 633 nm, Em above 650 nm. Magnification: ×200. Both Na_2_S and SDSS significantly increased the fluorescence intensity.

On the basis of these findings, we synthesized SDSS (**Figure 1A**), a hybrid compound derived from Ac-DSS and ADT-OH (a proven H_2_S-releasing moiety). We hypothesized that H_2_S-releasing SDSS molecule may protect osteoblasts from oxidant-stress-induced cell injury and stimulate cell differentiation.

## Materials and Methods

### Chemicals and Reagents

DSS was purchased from Baoji Herbest Bio-Tech Co., Ltd (Baoji, Shanxi, China). Other chemicals: NaHS, Na_2_S (for probe detect H_2_S, we use Na_2_S as H_2_S donor), methylthiazolyltetrazolium (MTT), Hoechst 33342, LY294002, 2′, 7′ – H_2_DCFDA and Glutathione Assay Kit were purchased from Sigma–Aldrich (St. Louis, MO, USA). Primary antibodies against phosphorylated extracellular- signal-regulated kinase 1/2 (ERK1/2), phosphorylated p38, phosphorylated c-Jun N-terminal kinase (JNK), phosphorylated Akt, and total ERK1/2, p38, JNK and Akt were purchased from Cell Signaling Technology (Beverly, MA, USA). The superoxide dismutase (SOD) assay kit was purchased from Cayman Chemical (Ann Arbor, MI, USA). The iScriptTM complementary DNA (cDNA) synthesis kit was purchased from Bio-Rad Laboratories (Singapore).

### Experimental Animals

Adult Sprague Dawley rats (180–220 g) and new born Sprague Dawley rats were used for evaluation of SDSS metabolism in plasma and primary osteoblast culture respectively. Both protocols were approved by Institutional Animal Care and Use Committee of Xi’an Jiaotong University.

### Synthesis of SDSS

The chemical structure of SDSS is shown in **Figure 1A**. The steps involved in SDSS synthesis was as follow:

(1) Synthesis of ADT-OH: ADT-OH was prepared according to Li et al. (2007). Briefly, Anethole (80 mmol) was mixed with sulfur (560 mmol) in 40 ml of dimethylacetamide and heated at 145°C for 6 h. After incubation overnight at room temperature, unreacted sulfur was filtered and the solution was diluted with water (400 ml) to precipitate anethole dithiolethione sulphoraphane (ADT). The product was washed with ether and recrystallized from ethyl acetate to obtain ADT as dark orange needle-shaped crystals (13.789 g, 72%). ADT (3 g) and pyridine hydrochloride (15 g) was then mixed and heated at 215°C under N_2_ for 25 min. After cooling, HCl (1 M) in excess was added to the solution, and the precipitate was filtered and washed with water. After drying, the residue was purified by silica gel column chromatography (CH_2_Cl_2_ as eluant) to give ADT-OH (1.418 g, 50%). The maximal absorbance peak of ADT-OH was at 349 nm. ^1^H NMR: δ 7.59 (d, *J* = 9.0 Hz, 2H), 7.39 (s, 1H), 6.93 (d, *J* = 9.0 Hz, 2H). Electrospray ionization mas spectrometry: *m/z* calcd for [M-H]^-^ C_9_H_5_S_3_O, 225.0, found 225.1.
(2) Synthesis of Ac-DSS: DSS (2.0 g, 10 mmol) was added to a mixed solution of acetic anhydride (16 ml) and pyridine (1 ml), and the resulting mixture was stirred overnight at room temperature. The reaction mixture was poured into ice-water and acidized with 1M HCl to pH 2. The product was extracted with ethyl acetate three times, and the combined organic layers were washed with water and brine, and then finally dried over Na_2_SO_4_. The organic solvent was evaporated under reduced pressure, and the yellow oil residue was purfied by column chromatography on silica gel and eluted with CH_2_Cl_2_/CH_3_OH (v/v, 98:2) to give a white waxy Ac-DSS (1.688 g, 52%). ^1^H NMR: δ 7.13 -7.09 (m, 3H), 5.23-5.19 (dd, *J* = 9.0, 6.0 Hz, 1H), 3.24-3.18 (dd, *J* = 15.0, 6.0 Hz, 1H), 3.14–3.07 (dd, *J* = 15.0, 9.0 Hz, 1H), 2.28 (s, 6H), 2.10 (s, 3H). Electrospray ionization mas spectrometry: *m/z* calcd for [M-H]^-^ C_15_H_15_O_8_, 323.1, found 322.9.
(3) Synthesis of SDSS: A solution of dicyclohexylcarbodiimide (2.57 mmol) in anhydrous dichloromethane (5 ml) was added dropwise to a solution containing Ac-DSS (2.58 mmol), ADT-OH (2.15 mmol), and 4-dimethylaminopyridine (0.39 mmol) in dichloromethane (15 ml) at 0°C under N_2_. The mixture was stirred for 1.5 h at room temperature. After the white dicyclohexylurea had been filtered, the filtrate was washed with 1 M HCl (20 ml), H_2_O (20 ml) and NaHCO_3_ (aqueous saturated, 20 ml) and brine consecutively. The organic solution was evaporated under reduced pressure, and the residue was purified by reversed-phase chromatography (RP-18, H_2_O/ACN, v/v, 60:40–50:50) to give SDSS (851 mg, 71%) as an orange solid (Sparatore et al., 2009). The maximal absorbance peak of SDSS was at 318 nm. ^1^H NMR: δ 7.64 (d, *J* = 9.0 Hz, 2H), 7.39 (s, 1H), 7.19-7.15 (m, 3H), 7.04 (d, *J* = 9.0 Hz, 2H), 5.36 (t, *J* = 6.0 Hz, 1H), 3.28 (d, *J* = 6.0 Hz, 2H), 2.31 (s, 3H), 2.30 (s, 3H), 2.18 (s, 3H). Electrospray ionization mas spectrometry: *m/z* calcd for [M+H]^+^ C_24_H_21_S_3_O_8_, 533.0, found 533.0.

^1^H NMR spectra were recorded with an AC300 spectrometer (Bruker, Karlsruhe, Germany) at 300 MHz, using CDCl_3_ as solvent. The electrospray ionization mass spectra were obtained with an LCQ ion trap mass spectrometer (Finnigan MAT, San Jose, CA, USA) in negative electrospray ionization mode. Atmospheric pressure chemical ionization mass spectra were collected with a Brucker amaZonX mass spectrometer.

### HPLC Analyses of Drugs Metabolites

Metabolites of ADT-OH and SDSS *in vivo* experiments were detected by a Waters HPLC system (Milford, MA, USA) with an Alliance 2659 separation module, a 2996 photodiode array (PDA) detector (Giustarini et al., 2010b). Briefly, Sprague-Dawely rats were anesthetized with Ketamine/xylazine (75/10 mg/kg) mixture. SDSS or ADT-OH (30 mg/kg) was injected in the lateral tail vein (Yu et al., 2014). Blood samples were collected through cardiac puncture 5, 15, and 30 min after injection. 20 μl of plasma were added to 60 μl of HPLC grade ACN and vigorously shaken for 30 s. After centrifugation, 50 μl supernatant were added with 5 μl of 1% (v/v) TFA. The resulting samples were load onto an HPLC column apparatus (Waters C18 column, 4.6 mm × 250 mm, 5 μm, Atlantis, Ireland) and separated by applying a mixture of a 0.05% (v/v) TFA/H_2_O (phase A) and ACN (phase B), gradient: 0–30 min, 64% ACN; 30–45 min, 90% ACN; 45–50 min, 90% ACN; 50–65 min, 64% ACN; 65–85 min, 64% ACN. The flow rate was 6.3 ml/min and the injection volume was 20 μl. The identity of ADT-OH was determined by analyzing plasma samples, spiked with additional solutions of ADT-OH in ACN. The UV detector was recorded at 330 nm.

### SDSS-Releasing H_2_S Assay

To image the release of H_2_S from SDSS, a highly selective and sensitive H_2_S probe 8 was used (Wu et al., 2014). Briefly, MC3T3-E1 cells were seeded on a four-well glass chamber slide (Lab-Tek chambered no. 1.0 borosilicate coverglass system) and incubated at 37°C in 5% CO_2_ for 24 h. The cell culture medium was then discarded, and 0.7 ml working medium containing the probe 8 was added. After 2 h incubation, cells were washed with phosphate-buffered saline (PBS) three times to remove the excess probe, and then treated with SDSS or Na_2_S for 3 h. Fluorescence images were taken with a confocal microscopy (Olympus IX 81, Fluroview FV1000). The H_2_S probe 8 was excited at 633 nm, and the emission above 650 nm was collected.

### Cell Culture and Treatment

The murine calvaria-derived MC3T3-E1 osteoblast-like cell line was purchased from the American Type Culture Collection (Manassas, VA, USA). Cells were cultured at 37°C in a humidified atmosphere of 5% CO_2_ and 95% air with alpha minimum essential medium (α-MEM) supplemented with 10% fetal bovine serum (FBS) and 1% penicillin/streptomycin. This basic medium was replenished every 3 days. For cell differentiation, the cells were transferred into the medium supplemented with L-ascorbic acid and β-glycerol phosphate at a final concentration of 50 μg/ml and 5 mM respectively (Fatokun et al., 2006). Cells were seeded into 60 mm dishes and incubated until 70% confluence was reached. To detect the protection of SDSS against H_2_O_2_-induced cell injury and the underlying signaling mechanisms, MC3T3-E1 cells were pretreated with SDSS for 60 min, washed twice with PBS, and then subjected to H_2_O_2_.

### Primary Culture of Osteoblasts

Primary osteoblasts were isolated from the calvaria of 2- to 4-day-old Sprague Dawley rats. Briefly, calvaria were cut to 1-mm^2^ debris, then sequentially digested for 15 min in 1 × PBS that contained 0.2% type IV collagenase with EDTA to remove fibroblasts and for 45 min in 1 × PBS that contained 0.2% type IV collagenase to collect osteoblasts. Cells were expanded for 5–6 days in alpha minimum essential medium containing 10% FBS and plated at a density of 2.5 × 10^4^ cells per square centimeter.

### Cell Viability

Cell viability was evaluated with the MTT method. Briefly, cells were seeded in 96-well plates at approximately 1 × 10^4^/well and cultured overnight. The cells were pretreated with SDSS for 60 min and then incubated in freshly prepared medium containing 400 μM H_2_O_2_ for 4 h. The medium was removed at the end of treatment, and 200 μl fresh medium containing MTT at 0.5 mg/ml was added. After a further incubation at 37 °C for 4 h, the culture medium containing MTT was aspirated. Dimethyl sulfoxide (150 μl) was then added into each well, and the absorbance at 570 nm was measured using a spectrophotometric plate reader.

### Annexin V-Fluorescein Isothiocyanate/Propidium Iodide Double Staining Assay

The annexin V–fluorescein isothiocyanate (FITC)/propidium iodide (PI) apoptosis detection kit was purchased from 7 Sea Biotech (Shanghai, China). Annexin V–FITC/PI double staining assays were performed according to the manufacturer’s instructions. Briefly, cells in early and late apoptotic stages were quantified by the annexin V–FITC/PI double staining assay. Treated and untreated cells were harvested with trypsinization and washed twice with PBS. Then cells were resuspended in 500 μl binding buffer, followed by staining with 10 μl annexin V and 5 μl PI in the dark at room temperature for 15 min. The stained cells were examined immediately with a fluorescence-activated cell sorting flow cytometry analyzer with emission filters of wavelength 488–530 nm for green fluorescence of annexin V and 488–630 nm for red fluorescence of PI. At least 10,000 events per sample were acquired to ensure obtaining adequate data.

### Measurement of Cell Apoptosis With Hoechst 33342 Staining

To visualize nuclear morphology, cells were fixed in 4% paraformaldehyde for 5 min and stained with 2.5 μg/ml Hoechst 33342 DNA dye. Uniformly stained nuclei were scored as healthy, viable cells. Condensed or fragmented nuclei were scored as apoptotic. To obtain unbiased counting, petri dishes were coded, and cells were scored blindly without knowledge of their prior treatment.

### Caspase 3 Activity Assay

Caspase activities were measured with a caspase activity kit according to the manufacturer’s instructions (BioVision, Mountain View, CA, USA). Briefly, after different treatments, cells were washed with cold PBS, resuspended in lysis buffer, and left on ice for 15 min. The lysate was centrifuged at 16,000 *g* at 4 °C for 15 min. Activity of caspase 3 was measured with the use of the substrate peptide Ac-DEVDp-nitroanilide. We qualified the release of *p*-nitroanilide by determining the absorbance with a spectrophotometric plate reader at 405 nm. The fold increase in activity was calculated as the ratio between values obtained from treated samples versus those obtained from untreated controls.

### Alkaline Phosphatase Activity Assay and Collagen Content Assay

To measure the alkaline phosphatase (ALP) activity and collagen content, cells were pretreated with SDSS for 60 min and then incubated in freshly prepared medium containing 400 μM H_2_O_2_. Four hours later, H_2_O_2_ was washed out with PBS. Cells were cultured with fresh differentiation medium. After 5 days, the medium was collected for extracellular soluble collagen content assay by a Sircol collagen assay kit (Biocolor, Carrickfergus, UK). The monolayer cells were collected and lysed with cell lysis buffer and centrifuged at 12,000 *g* for 10 min. The supernatant was collected, and ALP activity and protein concentration were determined with an ALP activity assay kit (Cell Biolabs, San Diego, CA, USA) and a bicinchoninic acid protein assay kit (Bio-Rad, Hercules, CA, USA) respectively.

### Measurement of Extracellular Matrix Calcium Deposits

Staining with alizarin red S is used to visualize bone nodule formation and calcium deposition of osteoblasts cultured *in vitro* (Kim et al., 2012). Cells were pretreated with SDSS for 60 min and then incubated in freshly prepared medium containing 400 μM H_2_O_2_ for 4 h. Then H_2_O_2_ was washed out with fresh differentiation medium and the cells were cultured for 21 days. At harvest, MC3T3-E1 cells were fixed and stained with alizarin red S. Calcium precipitation was visualized by light microscopy.

### ROS Measurement

H_2_DCFDA was used to detect ROS production as described previously (Guo C. et al., 2014). Briefly, cells were seeded in black 96-well plates or 35-mm dishes and cultured overnight. Cells were treated with SDSS for 60 min and then incubated with H_2_O_2_ (400 μM) for 4 h. The culture medium was discarded and the cells were washed twice with PBS. The culture medium was replaced with phenol-red-free Dulbecco’s modified Eagle’s medium containing H_2_DCFDA (10 μM) for 30 min. ROS production was measured with a fluorescence microscope (Nikon) or a fluorescence reader (Safire2, Tecan Group) at an excitation wavelength of 490 nm and an emission wavelength of 520 nm.

### Superoxide Dismutase (SOD) Activity and Glutathione (GSH) Activity

GSH assays were performed according to the instructions of a glutathione assay kit (Sigma). Briefly, cells were suspended in PBS and centrifuged at 600 *g* to obtain packed cell pellets. Then 3 volumes of the 5% SSA solution were then added to cell pellets. Repeated freeze-thaw cycles were undertaken for thorough cell lysis. After centrifugation at 10,000 *g* for 10 min, 10 μl supernatants were added into individual wells, followed by addition of 150 μl working mixture (glutathione reductase and 5,5-dithiobis(2-nitrobenzoic acid) solution) and incubation for 5 min at room temperature. Then 50 μl NADPH solution was added to initiate the reaction. The GSH content was determined by kinetic measurement with 1-min intervals for 5 min at 412 nm and calculated by comparison with standards.

Superoxide dismutase activity was measured in cells using a Superoxide Dismutase Assay Kit (Cayman Chemicals, Inc, Ann Arbor, MI, USA). Briefly, cells were sonicated in 20 mM *N*-(2-hydroxyethyl)piperazine-*N*-ethanesulfonic acid buffer, pH 7.2, containing 1 mM ethylene glycol bis(2-aminoethyl ether)tetraacetic acid, 210 mM mannitol and 70 mM sucrose, on ice. After centrifugation, the supernatants were collected and 10 μl supernatants were added into individual wells. Reaction was initiated by addition of diluted xanthine oxidase to all wells, and then the plate was incubated on a shaker at room temperature for 20 min. The absorbance was read at 450 nm.

### Western Blotting Assay

Cells were washed twice with chilled PBS and lysed in radioimmunoprecipitation assay lysis buffer. Protein samples were separated by 12 % sodium dodecyl sulfate–polyacrylamide gel electrophoresis and transferred to a nitrocellulose membrane (GE Healthcare, Chalfont St Giles, UK). After blocking at room temperature in 10 % milk in 10 mM tris(hydroxymethyl)aminomethane-HCl, 120 mM NaCl, and 0.1 % Tween 20, pH 7.4 (TBST buffer) for 1 h, the membrane was incubated with various primary antibodies (1:1000 dilution) at 4°C overnight. Membranes were then washed three times in TBST buffer, followed by incubation with horseradish peroxidase conjugated anti-rabbit IgG (1:10,000 dilution) at room temperature for 1 h and washed three times in TBST buffer. Membranes were visualized with an enhanced chemiluminescence kit (GE Healthcare). Densitometric quantification was performed with ImageJ. The protein bands were quantified and normalized to the total protein concentration.

### Quantitative Reverse Transcription PCR

Cells were seeded in six-well plates and cultured for 24 h. After different treatments, cells were collected. Total RNA from each well was isolated separately with an RNeasy Mini kit (Qiagen, Valencia, CA, USA). RNA (1 μg) from each well was reverse transcribed separately into cDNA with an iScript cDNA synthesis kit (Bio-Rad, Hercules, CA, USA). Quantitative reverse transcription PCR (qRT-PCR) was performed with SYBR Green quantitative PCR master mix (Applied Biosystems). All amplifications were normalized by β-actin. Data were analyzed using the comparison Ct(2^-ΔΔCt^) method and expressed as fold change compared to respective control. The primer sequences used for the qRT-PCR assay (5′–3′) were as follows: β-actin forward, TGCGTGACATCAAAGAGAAG; β-actin reverse, GATGCCACAGGATTCCATA; runt-related transcription factor 2 (Runx2) forward, TTCTCCAACCCACGAATG CAC; Runx2 reverse, CAGGTACGTGTGGTAGTGAGT; bone sialoprotein (BSP) forward, GAATCCACATGCCTA TTGC; BSP reverse, AGAACCCACTGACCCATT; osteocalcin forward, GAACAGACTCCGGCGCTA; osteocalcin reverse, AGGGAGGATCAAGTCCCG; osterix forward, TGGCCATGCTGACTGCAGCC; osterix reverse, TGGGT AGGCGTCCCCCATGG; heme oxygenase 1 (HO-1) forward, CCAGGCAGAGAATGCTGAGTTCATG; HO-1 reverse, TGCAGCTCTTCTGGGAAGTAGACAG; glutamate-cysteine ligase modifier subunit (GCLM) forward, CCCAGATTTGGTCAGGGAGTTTCCA; GCLM reverse, ACTGAACAGGCCATGTCAACTGCA.

### Luciferase Activity

ARE-Luc reporter plasmids were purchased from Yeasen Biotechnology (Shanghai, China). The MC3T3-E1 cells, cultured in a 24-well plate, were transfected with 100 ng pARE-Luc-Neo plasmid and 20 ng pCMV-β-gal by means of the FuGENE 6 protocol (Roche Molecular Biochemicals). The day after transfection, the medium was replaced with fresh medium. Cells were cultured for an additional 12 h before drug treatment. Then the cells were treated with SDSS or DSS. After treatment, cells were washed twice with ice-cold PBS and harvested in reporter lysis buffer. The β-galactosidase activity was determined as described previously (Marshall, 1995). The luciferase activity was determined according to the protocol provided by the manufacturer (Promega, Madison, WI, USA).

### Statistical Analysis

The data are expressed as the mean ± the standard error of the mean. Comparison of multiple groups was performed by one-way analysis of variance followed by Tukey’s *post hoc* test. A probability level of less than 0.05 was used to establish significance.

## Results

### SDSS Metabolism in Rat Plasma

The levels and formation of metabolites of SDSS were evaluated in rat plasma 5–30 min after administration of the SDSS (30 mg/kg) or ADT-OH (30 mg/kg). As shown in **Figure 1B**, SDSS was rapidly deacetylated, yielding deacetylated metabolite which, in turn, was hydrolyzed to produce dithiolethione moiety (ADT-OH) and DSS. SDSS was also de-esterificated and generated concomitantly ADT-OH and Ac-DSS. Moreover, the metabolism of the deacetylation and de-esterification products was completed within 5 min as evidenced by the rapid disappearance of these products accompanied by the appearance of ADT-OH and its metabolites. The retention time of ADT-OH was at 10.7 min. This was proved by plasma samples spiked with additional solution of ADT-OH and the peak area was evidently enhanced (**Figure 1B**). The retention time of metabolites 1 and 2 was 5.1 min and 6.6 min, respectively. Peaks 1 and 2 are the metabolites of ADT-OH, (a, b, or c) shown in **Figure 1C**. The retention time of the final metabolite at 6.6 min reached the maximal level at 15 min after the administration of SDSS and decreased relatively slower over time. The last metabolic degradation of SDSS [**Figures 1B**(e–g)] was similar to that of ADT-OH [**Figures 1B** (a–c)]. The release of SDSS was further proved by plasma samples spiked with additional solution of ADT-OH and the peak area was evidently enhanced [**Figures 1B**(d,h)]. According to the previous reports, ADT-OH was found to release H_2_S at different periods from the three sulfur atoms of the trithione moiety (Sparatore et al., 2009; Giustarini et al., 2010a). We therefore speculate that SDSS containing a dithiolethione moiety also releases H_2_S (**Figure 1C**).

### SDSS Releases H_2_S in MC3T3-E1 Cells

We further detected H_2_S released from SDSS with a fluorescent probe 8, a highly selective and sensitive H_2_S probe which takes advantage of Cu(II)-cyclen complex as the quencher of fluorescence and the reaction center for H_2_S; once reacted with H_2_S, it can fluoresce up to 20 folds more. As shown in **Figure 1D**, after treatment with 100 μM inorganic H_2_S donor Na_2_S, the MC3T3-E1 cells showed large fluorescent signals compared to that of control cells, which indicates probe 8 can detect H_2_S well in MC3T3-E1 cells. SDSS at both 50 and 100 μM also significantly increased the fluorescence intensities in cells, confirming that SDSS released H_2_S in MC3T3-E1 cells.

### SDSS Alleviates H_2_O_2_-Induced MC3T3-E1 Cell Injury

**FIGURE 2:**
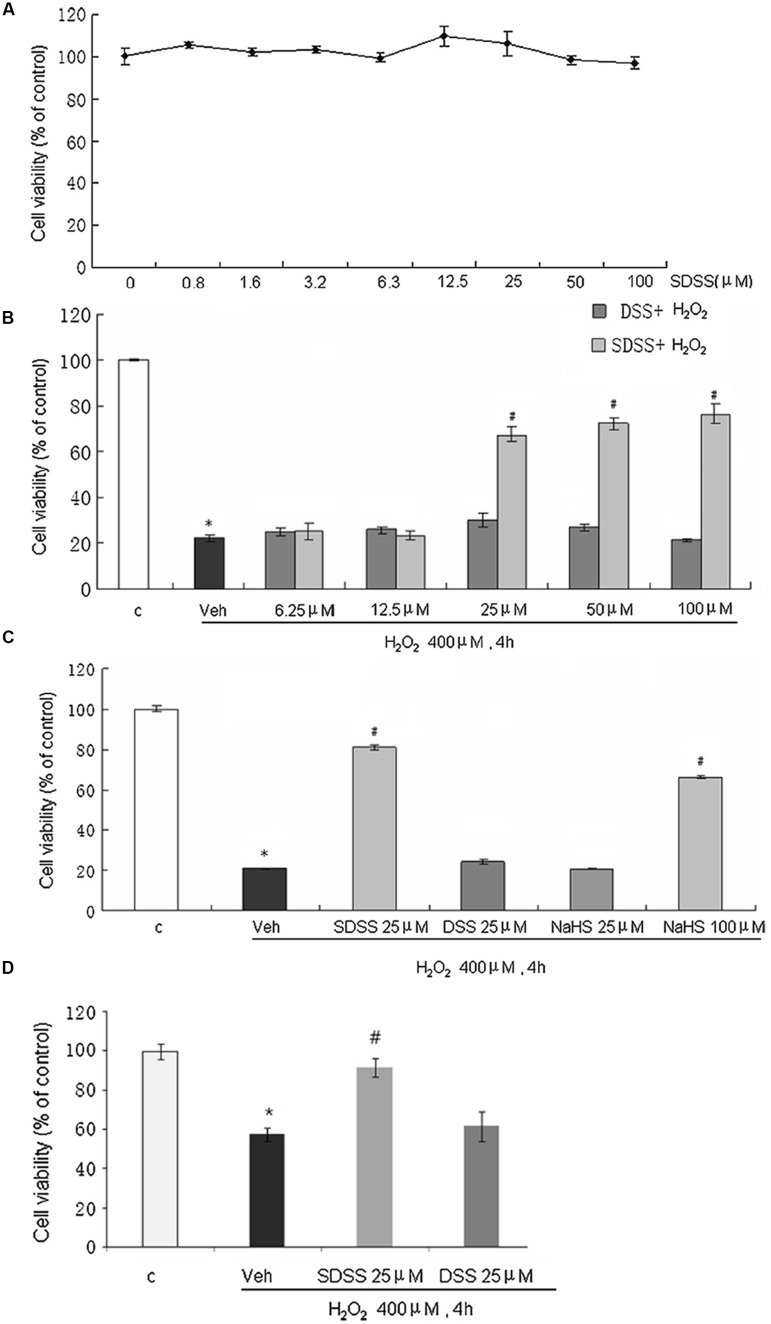
**Effects of SDSS on H_2_O_2_-induced cell injury in MC3T3-E1 osteoblastic cells.** Cell viability was determined by the methylthiazolyltetrazolium assay. **(A)** Effect of SDSS (0–100 μM) alone. **(B)** Concentration-dependent effect of SDSS and DSS against H_2_O_2_-induced cell injury. **(C)** Comparison of the effect of DSS, SDSS, and NaHS on H_2_O_2_-treated cells (400 μM, 4 h). **(D)** Effect of SDSS on viability of primary cultured rat osteoblasts in cells treated with H_2_O_2_ for 4 h. Mean ± standard error of the mean, *n* = 4–8. ^∗^*p* < 0.05 versus the control groups (c); #*p* < 0.05 versus the H_2_O_2_ groups; veh, vehicle.

Treatment with SDSS at a concentration range from 1 to 100 μM for 30 h did not significantly affect MC3T3-E1 cell viability (**Figure 2A**). Interestingly, SDSS at 25 μM or higher produced significant protective effects against H_2_O_2_-induced cell injury (**Figure 2B**). Both DSS and NaHS at 25 μM did not produce significant beneficial effects (**Figure 2C**). At 100 μM, NaHS produced a comparable protective effect compared with that caused by SDSS at 25 μM. The protective effect of SDSS was further confirmed with the primary cultured rat osteoblasts. Pretreatment with 25 μM SDSS reversed the decreased cell viability from 57.6% in H_2_O_2_ treatment group to 91.7% (**Figure 2D**).

### SDSS Inhibits H_2_O_2_-Induced Apoptosis in MC3T3-E1 Cells

**FIGURE 3:**
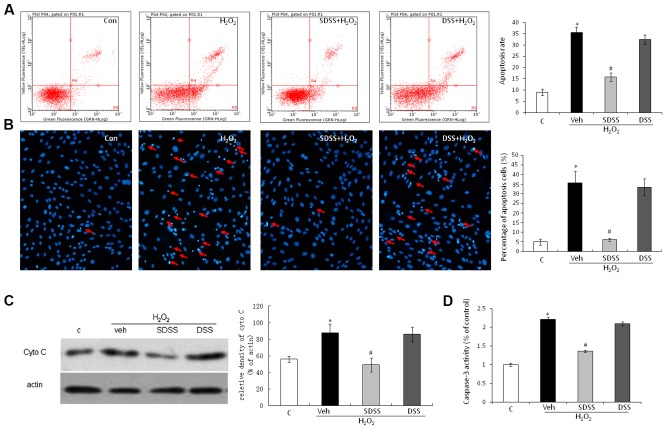
**Effect of SDSS on H_2_O_2_-induced cell apoptosis. (A)** Cell apoptosis was detected by annexin V–fluorescein isothiocyanate/propidium iodide double staining and examined with a fluorescence-activated cell sorting flow cytometry analyzer. **(B)** Cell apoptosis was detected by Hoechst 33342 staining in cells treated with vehicle, H_2_O_2_, H_2_O_2_+ DSS (25 μM) and H_2_O_2_ + SDSS (25 μM). Cells with condensed or fragmented nuclei were identified as apoptosis cells and counted based on nuclear condensation or fragmentation. **(C)** Representative Western blots and group data for cytochrome c cytosolic expression. Pretreatment with SDSS (25 μM, 1 h) significantly inhibited H_2_O_2_ induced release of cytochrome c. **(D)** Caspase 3 activity in differently treated cells. SDSS reduced the H_2_O_2_-induced increased caspase 3 activity. Mean ± standard error of the mean, *n* = 4–8. ^∗^*p* < 0.05 versus control (c); #*p* < 0.05 versus H_2_O_2_ group; cyto, cytochrome; DSS, danshensu; veh, vehichle.

Both Annexin V-FITC/PI double staining (**Figure 3A**) and Hoechst 33342 staining assays (**Figure 3B**) were performed to quantify the apoptosis of MC3T3-E1 cells. As presented in **Figures 3A,B**, H_2_O_2_ triggered a higher magnitude of apoptosis relative to the control. After treatment with SDSS (25 μM), the percentage of apoptotic cells dropped significantly. This was not observed in cells treated with DSS (25 μM). Cytochrome c release is an obvious phenomenon in the early period of apoptosis. Western blotting analysis also showed that H_2_O_2_ induced cytochrome c release and SDSS inhibited this effect (**Figure 3C**). In addition, SDSS also decreased the H_2_O_2_-stimulated caspase 3 activity (**Figure 3D**). These results suggest that SDSS, but not DSS, produced a significant antiapoptotic effect.

### SDSS Stimulates Osteoblast Differentiation under Normal and Oxidant Conditions

**FIGURE 4:**
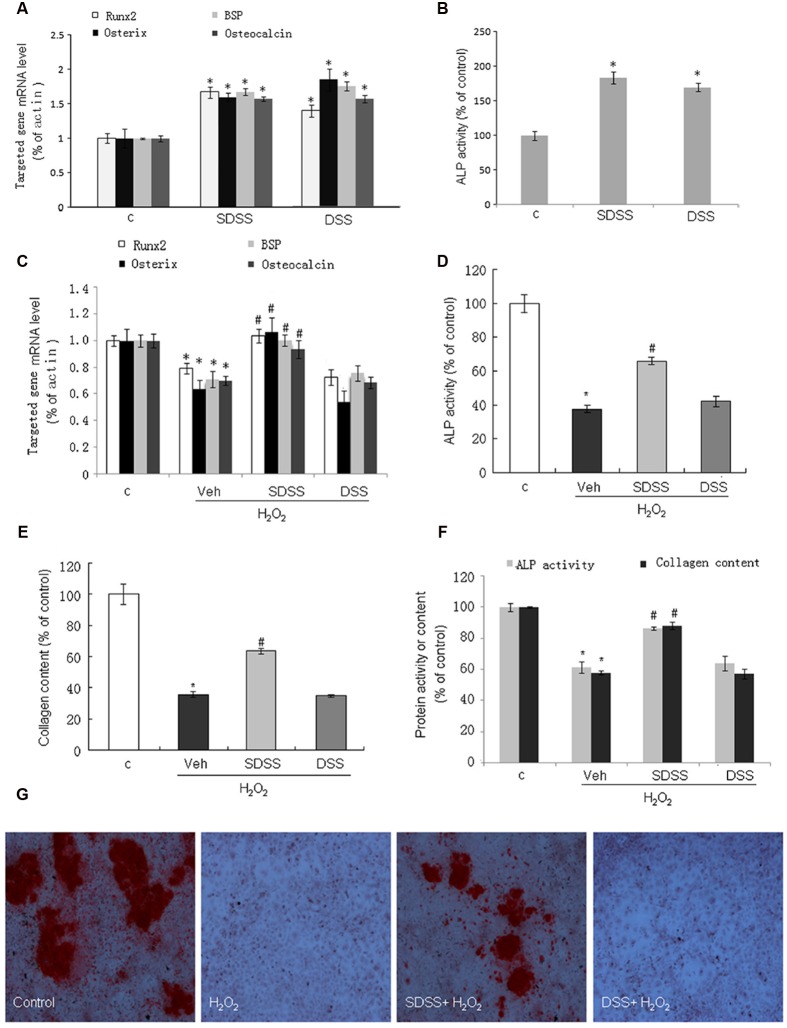
**Effect of SDSS on MC3T3-E1 cell differentiation. (A,C)** Quantitative reverse transcription PCR analysis of the messenger RNA (mRNA) levels of bone sialoprotein (BSP), runt-related transcription factor 2 (Runx2), osterix, and osteocalcin in the absence **(A)** or presence **(C)** of H_2_O_2_. **(B,D)** Effect of SDSS and DSS on alkaline phosphatase (ALP) activity in cells treated without **(B)** and with **(D)** H_2_O_2_. **(E)** SDSS but not DSS attenuated H_2_O_2_ down-regulated collagen expression in MC3T3-E1 cells on day 5 after H_2_O_2_ treatment. **(F)** Effect of SDSS and DSS on ALP activity and collagen expression in the primary cultured osteoblasts on day 5 after H_2_O_2_ treatment. **(G)** Representative image of mineralized bone nodule formation. H_2_O_2_ decreased bone nodule formation and SDSS alleviated the effect of H_2_O_2_. Mean ± standard error of the mean, *n* = 4-8. ^∗^*p* < 0.05 versus control (c); #*p* < 0.05 versus H_2_O_2_ group.

We first investigated the effects of SDSS and DSS on osteoblast differentiation under normal condition. Both SDSS and DSS (25 μM, 18 h) stimulated BSP, osteocalcin, osterix, and Runx2 mRNA expression (**Figure 4A**) and ALP activity (**Figure 4B**). This is consistent with the previous reports that DSS can stimulate osteoblast differentiation (Cui et al., 2009; Guo Y. et al., 2014). Treatment with H_2_O_2_ (400 μM) for 4 h markedly reduced mRNA levels of BSP, osteocalcin, osterix, and Runx2. Pretreatment with SDSS, but not DSS (25 μM, 1 h), reversed these effects (**Figure 4C**). ALP activity and collagen expression were largely reduced in both MC3T3-E1 cells (**Figures 4D,E**) and primary cultured osteoblasts (**Figure 4F**) on the fifth day after the short-term treatment with H_2_O_2_ (400 μM, 4 h). Pretreatment with SDSS but not DSS reversed these effects. Alizarin red S staining showed that pretreatment with SDSS reversed H_2_O_2_-suppressed bone nodule formation (**Figure 4G**). These data suggest that SDSS may restore osteoblast differentiation function during oxidative stress.

### SDSS Produces Anti-oxidant Effects in MC3T3-E1 Cells

**FIGURE 5:**
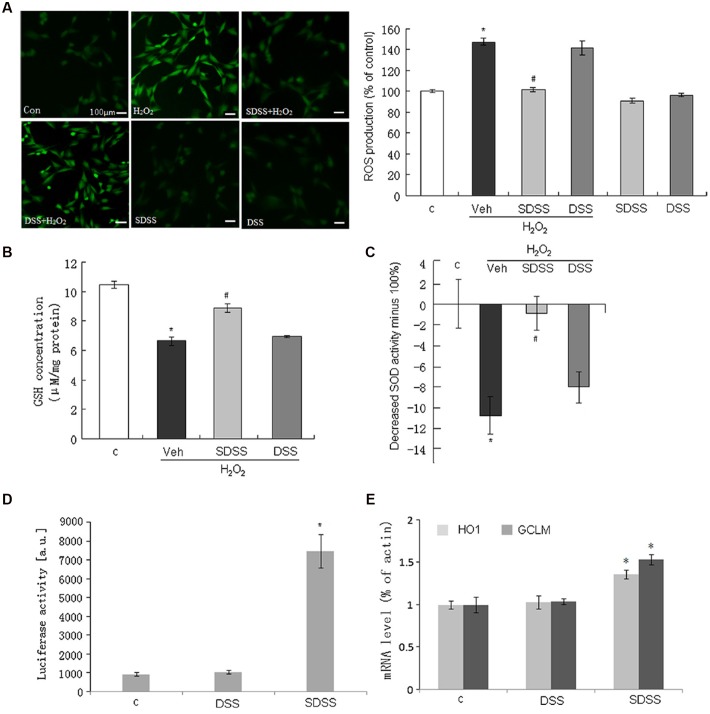
**Effect of SDSS on the production of reactive oxygen species (ROS) and the activities of antioxidants. (A)** Representative images (left) and group data (right) showing that SDSS, but not DSS, suppressed H_2_O_2_-induced ROS production. **(B,C)** SDSS, but not DSS, reversed H_2_O_2_-reduced GSH **(B)** and SOD **(C)** activity. **(D,E)** SDSS, but not DSS, stimulated antioxidant response element-luciferase activity **(D)** and quantitative reverse transcription PCR analysis of the messenger RNA (mRNA) level of heme oxygenase 1 (HO1) and glutamate-cysteine ligase modifier subunit (GCLM) **(E)**. Mean ± standard error of the mean, *n* = 4–8. ^∗^*p* < 0.05 versus the control group; #*p* < 0.05 versus the H_2_O_2_ group; c, control; DSS, danshensu; Veh, vehicle group.

We further determined the anti-oxidant effect of SDSS. As shown in **Figure 5A**, pretreatment with SDSS (25 μM, 60 min) alleviated H_2_O_2_-induced ROS accumulation. We also studied the involvement of GSH and SOD activity. As expected, pretreatment with SDSS but not DSS reversed H_2_O_2_ reduced intracellular GSH concentration and SOD activity (**Figures 5B,C**). The ARE-Luc reporter assay showed that SDSS was a potent ARELuc inducer (**Figure 5D**). HO-1 and glutamate–cysteine ligase [comprising a catalytic subunit (GCLC) and a modifier subunit (GCLM)] are ARE-dependent genes. SDSS (25 μM, 18 h) enhanced HO-1 and GCLM mRNA expression levels (**Figure 5E**), but DSS (25 μM, 18 h) failed to produce the similar effects. These results imply that the antioxidant effect of SDSS may not solely come from DSS.

### Involvement of MAPKs and the Phosphatidylinositol 3-Kinase (PI3K)/Akt Pathway in the Protective Effect of SDSS

**FIGURE 6:**
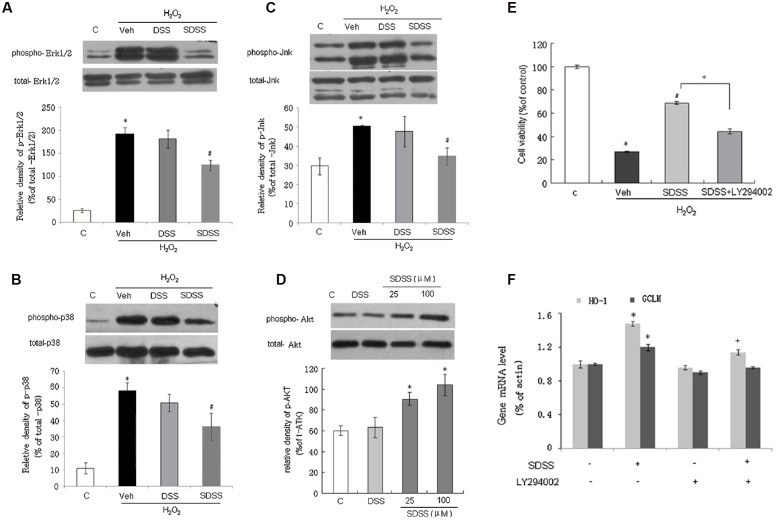
**Involvement of mitogen-activated protein kinases and the phosphatidylinositol 3-kinase/Akt pathway in the protective effect of SDSS.** Representative Western blots and group data showing the effect of SDSS and DSS on H_2_O_2_-stimulated extracellular signal-regulated kinase 1/2 (Erk1/2; **A**), p38 **(B)**, and c-Jun N-terminal kinase (Jnk; **C**) phosphorylation. **(D)** Representative Western blots and group data showing that SDSS (25 and 100 μM) induced Akt phosphorylation. **(E,F)** LY294002 (15 μM, 15 min) attenuated the effect of SDSS (25 μM, 18 h) on cell viability **(E)**, and messenger RNA (mRNA) expression levels of heme oxygenase 1 (HO-1) and glutamate-cysteine ligase modifier subunit (GCLM) **(F)**. Mean ± standard error of the mean, *n* = 4–6. ^∗^*p* < 0.05 versus control group; #*p* < 0.05 versus the H_2_O_2_ group; +*p* < 0.05 versus the SDSS group; c, control; DSS, danshensu; Veh, vehicle group.

We further examined the involvement of MAPKs and PI3K/Akt signaling pathway in the effects of SDSS. As shown in **Figures 6A–C**, SDSS pretreatment significantly attenuated H_2_O_2_-induced phosphorylation of ERK1/2, p38, and JNK. These effects were in accordance with the findings of our previous reports that H_2_S protects MC3T3-E1 osteoblasts against H_2_O_2_-induced oxidative damage by inhibition of p38 and ERK1/2 protein kinases. Pretreatment of MC3T3-E1 cells with SDSS alone stimulated Akt phosphorylation in a concentration-dependent manner (**Figure 6D**). Inhibition of the PI3K/Akt signaling pathway with LY294002 (15 μM, 15 min; a selective PI3K/Akt inhibitor) reversed the protective effect of SDSS on H_2_O_2_-induced cell injury (**Figure 6E**), and abolished the stimulatory effect of SDSS on HO-1 and GCLM mRNA expression (**Figure 6F**).

## Discussion

The H_2_S-releasing property of SDSS was first examined in this study. SDSS can be rapidly deacetylated or de-esterificated to generate ADT-OH after injection into animals. The retention time of ADT-OH was about 10 min. ADT-OH can release H_2_S from the three sulfur atoms of the trithione moiety (Sparatore et al., 2009; Giustarini et al., 2010a). We also further confirmed the release of H_2_S from SDSS in MC3T3-E1 cells by using a novel and sensitive H_2_S probe 8. SDSS increased fluorescence intensities obviously compared with the control group. These results suggest that SDSS is a novel H_2_S-releasing compound and can release H_2_S at a moderate rate.

After confirming the H_2_S releasing property of SDSS, we continued to study the biological functions of SDSS. Both NaHS and DSS have been reported to protect cells against apoptosis and oxidative stress (Pan et al., 2006; Sivarajah et al., 2006; Yong et al., 2008a; Xu et al., 2011; Li et al., 2012; Lu et al., 2012, 2014). However, the doses of DSS used to study its antioxidant effect are largely varied in different experiments. For instances, Lu et al. (2014) reported DSS produced significant effect at 1 μM, whereas Cui reported it can only produce a similar protective effect at 300 μM (Cui et al., 2013). The discrepancy in DSS concentrations used in different studies is probably due to the different sensitivities of tissues/preparations of the same tissue to DSS. In the present study, we found that DSS alone at 25 μM stimulated osteoblast differentiation in normal situation (without H_2_O_2_ insults), but failed to produce significant beneficial effects in H_2_O_2_-treated MC3T3-E1 cells. These data imply that higher concentration of DSS may be needed to overcome oxidative stress-induced MC3T3-E1 cell injury. Interestingly, even though neither DSS nor NaHS at 25 μM protected osteoblast cells, SDSS at the same concentration produced significant beneficial effects against H_2_O_2_-induced cell injury in MC3T3-E1 cells. Our data imply that the protective effects of SDSS may come from the additive effect from H_2_S and DSS and/or the long-lasting H_2_S- and DSS releasing property.

Oxidative stress not only induced osteoblastic cell apoptosis, but also impeded osteoblasts differentiation (Liu et al., 2012; Yang et al., 2013). We further investigated the effects of SDSS on MC3T3-E1 cell differentiation. BSP, Runx2, osterix, and osteocalcin are markers representing the osteoblast differentiation phenotype. The mRNA levels of these markers were significantly suppressed in H_2_O_2_-treated MC3T3-E1 cells, and this suppression was reversed by SDSS pretreatment. These data imply that SDSS promotes osteoblast differentiation. We further confirmed this finding by measurement of collagen expression, ALP activity, and bone nodule formation. SDSS also prevented the reduction of ALP activity, the loss of collagen expression, and the inhibition of bone nodule formation in osteoblasts treated with H_2_O_2_. H_2_O_2_ impeded osteoblast differentiation, and SDSS restored the differentiation ability of osteoblasts under oxidant stress.

SDSS exerted protective effects by decreasing H_2_O_2_-induced ROS production. Depletion of GSH and inhibition of SOD activity result in accumulation of ROS and accelerate tissue injury (Sikka, 1996). SDSS stimulated SOD activity and promoted GSH production. Both HO-1 and glutamate-cysteine ligase (including the subunits GCLC and GCLM) are ARE-dependent genes and important factors against oxidant stress (Satoh et al., 2013). SDSS induced ARE activation. The qRT-PCR results revealed the SDSS effect of increasing HO-1 and GLCM expression. These results partly explained the underlying mechanisms for the SDSS effects on ROS scavenging. Mitochondrial dysfunction is also an important target and source of ROS, and is characterized by an increase in membrane permeability, which caused release of cytochrome c. Mitochondrial dysfunction participates in aging-related disease, including osteoporosis (Zhang et al., 2014; Lane et al., 2015). Our data show that cytosolic cytochrome c accumulation induced by H_2_O_2_ was significantly alleviated after pretreatment with SDSS. These findings indicate that SDSS could overcome oxidative stress and improve mitochondrial function.

Numerous studies have demonstrated that H_2_O_2_-induced apoptosis is mediated by activation of MAPKs (Park et al., 2005). Activated MAPKs participate in cell proliferation, differentiation, and cell death (Marshall, 1995; Davies et al., 2000). Our group reported that H_2_S protects osteoblasts from H_2_O_2_-induced cell injury by decreasing activation of H_2_O_2_-induced MAPKs (Xu et al., 2011). As SDSS releases H_2_S, we also investigated MAPKs in this study. As expected, SDSS significantly attenuated H_2_O_2_-induced p38, ERK1/2, and JNK phosphorylation. Apoptosis is one of the major outcomes of MAPK activation caused stressors. MAPKs initiate the mitochondrial apoptotic pathway because of enhanced pro-apoptotic protein activation. Previous studies have also reported that activation of p38 MAPK reduces SOD activity in several tissues (Banerjee Mustafi et al., 2009). SDSS may reverse H_2_O_2_-induced cell injury and decrease SOD activity through inhibition of the MAPKs pathways.

The PI3K/Akt signaling is an important pathway involved in cell differentiation (Tiong et al., 2010; Xie et al., 2012). In osteoporosis model rats, protein expression of phosphorylated PI3K and phosphorylated Akt in bone tissue was decreased dramatically (Xi et al., 2015). Akt activation promotes osteogenic differentiation (Feng et al., 2015). We therefore examined whether the PI3K/Akt signaling pathway is involved in the protective effects of SDSS. Unlike the MAPK pathway, the PI3K/Akt signaling pathway was significantly activated by SDSS pretreatment. Inhibition of PI3K/Akt pathway attenuated the effects of SDSS on oxidative-stress-induced cell injury and on HO-1 and GCLM mRNA expression. Previous studies have demonstrated that PI3K/Akt signaling plays an important role in expression of antioxidant enzymes, including HO-1, GCLC, and GCLM (Deng et al., 2013; Li et al., 2013). SDSS may also reverse H_2_O_2_-impaired GSH content via PI3K/Akt activation and increased expression of antioxidant enzymes.

## Conclusion

Our results show that SDSS, a new synthetic H_2_S-releasing DSS derivative, protected MC3T3-E1 osteoblasts against oxidative-stress-induced cell injury, apoptosis, and arrest of differentiation. This is mediated by its antioxidant effect through inhibition of MAPKs and activation of PI3K/ Akt pathways. Our data imply that SDSS has the potential to be developed as a drug to treat osteoporosis. However, long-term pharmacological and toxicological studies in animals are necessary before any conclusion can be made.

## Author Contributions

Participated in research design: XY, HW, DH, and J-SB. Conducted experiments: XY, HW, FH, ZW, and DL. Contributed new reagents or analytic tools: YY and HS. Performed data analysis: XY, HW, and ZW. Wrote or contributed to the writing of the manuscript: XY, HW, ZW, and J-SB.

## Conflict of Interest Statement

The authors declare that the research was conducted in the absence of any commercial or financial relationships that could be construed as a potential conflict of interest.

## Abbreviations

3-MST: 3-mercaptopyruvate sulfurtransferase
ADT-OH: 5-(4-hydroxyphenyl)-3H-1,2-dithiole-3-thione
Ac-DSS: α, 3, 4-tris (acetyloxy)-benzenepropanoic acid
CBS: Cystathionine-β-synthase
CSE: cystathionine γ-lyase DSS
Danshensu: H_2_DCFDA, Dichlorodihydrofluorescein diacetate
rMSCs: rat bone marrow stromal cells
SDSS, α-3: 4-tris (acetyloxy)-benzenepropanoic acid 4-(3-thioxo-3H-1, 2-dithiol-5-yl) phenyl ester.

## References

[B1] Bai X. C. Lu D. Bai J. Zheng H. Ke Z. Y. Li X. M. (2004). “Oxidative stress inhibits osteoblastic differentiation of bone cells by ERK and NF-kappaB. *Biochem. Biophys. Res. Commun.* 314 197–207. 10.1016/j.bbrc.2003.12.073 14715266

[B2] Banerjee Mustafi S. Chakraborty P. K. Dey R. S. Raha S. (2009). Heat stress upregulates chaperone heat shock protein 70 and antioxidant manganese superoxide dismutase through reactive oxygen species (ROS), p38MAPK, and Akt. *Cell Stress Chaperones* 14 579–589. 10.1007/s12192-009-0109-x 19291423 PMC2866949

[B3] Basu S. Michaelsson K. Olofsson H. Johansson S. Melhus H. (2001). Association between oxidative stress and bone mineral density. *Biochem. Biophys. Res. Commun.* 288 275–279. 10.1006/bbrc.2001.5747 11594785

[B4] Cooper C. (1999). Epidemiology of osteoporosis. *Osteoporos*. *Int.* 9(Suppl. 2), S2–S8. 10.1007/PL00004156 10525719

[B5] Cui G. Shan L. Hung M. Lei S. Choi I. Zhang Z. (2013). A novel Danshensu derivative confers cardioprotection via PI3K/Akt and Nrf2 pathways. *Int. J. Cardiol.* 168 1349–1359. 10.1016/j.ijcard.2012.12.012 23290949

[B6] Cui L. Liu Y. Y. Wu T. Ai C. M. Chen H. Q. (2009). “Osteogenic effects of D+beta-3,4-dihydroxyphenyl lactic acid (salvianic acid A, SAA) on osteoblasts and bone marrow stromal cells of intact and prednisone-treated rats. *Acta Pharmacol. Sin.* 30 321–332. 10.1038/aps.2009.9 19262556 PMC4002398

[B7] Davies S. P. Reddy H. Caivano M. Cohen P. (2000). “Specificity and mechanism of action of some commonly used protein kinase inhibitors. *Biochem. J.* 351(Pt 1), 95–105. 10.1042/0264-6021:3510095 10998351 PMC1221339

[B8] Deng X. Rui W. Zhang F. Ding W. (2013). PM2.5 induces Nrf2-mediated defense mechanisms against oxidative stress by activating PIK3/AKT signaling pathway in human lung alveolar epithelial A549 cells. *Cell Biol. Toxicol.* 29 143–157. 10.1007/s10565-013-9242-5 23525690

[B9] Fatokun A. A. Stone T. W. Smith R. A. (2006). Hydrogen peroxide-induced oxidative stress in MC3T3-E1 cells: Tthe effects of glutamate and protection by purines. *Bone* 39 542–551. 10.1016/j.bone.2006.02.062 16616712

[B10] Feng J. Sun Q. Liu L. Xing D. (2015). “Photoactivation of TAZ via Akt/GSK3beta signaling pathway promotes osteogenic differentiation.” *Int*. *J. Biochem. Cell Biol.* 66 59–68. 10.1016/j.biocel.2015.07.002 26159930

[B11] Giustarini D. Del Soldato P. Sparatore A. Rossi R. (2010a). “Modulation of thiol homeostasis induced by H2S-releasing aspirin. *Free Radic. Biol. Med.* 48 1263–1272. 10.1016/j.freeradbiomed.2010.02.014 20171274

[B12] Giustarini D. Perrino E. Tazzari V. Rossi R. (2010b). HPLC determination of novel dithiolethione containing drugs and its application for in vivo studies in rats. *J. Chromatogr. B Analyt. Technol. Biomed. Life Sci.* 878 340–346. 10.1016/j.jchromb.2009.11.047 20006565

[B13] Guo C. Liang F. Shah Masood W. Yan X. (2014). “Hydrogen sulfide protected gastric epithelial cell from ischemia/reperfusion injury by Keap1 s-sulfhydration, MAPK dependent anti-apoptosis and NF-kappaB dependent anti-inflammation pathway. *Eur. J. Pharmacol.* 725 70–78. 10.1016/j.ejphar.2014.01.009 24444438

[B14] Guo Y. Li Y. Xue L. Severino R. P. Gao S. Niu J. (2014). Salvia miltiorrhiza: an ancient Chinese herbal medicine as a source for anti-osteoporotic drugs. *J. Ethnopharmacol.* 155 1401–1416. 10.1016/j.jep.2014.07.058 25109459

[B15] Hu L. F. Lu M. Wong Hon P. T. Bian J. S. (2011). Hydrogen sulfide: neurophysiology and neuropathology. *Antioxid. Redox. Signal.* 15 405–419. 10.1089/ars.2010.3517 20812864

[B16] Isomura H. Fujie K. Shibata K. Inoue N. Iizuka T. Takebe G. (2004). Bone metabolism and oxidative stress in postmenopausal rats with iron overload. *Toxicology* 197 93–100. 10.1016/j.tox.2003.12.006 15003320

[B17] Jiang X. Lv B. Li P. Ma X. Wang T. Zhou Q. (2015). “Bioactivity-integrated UPLC/Q-TOF-MS of Danhong injection to identify NF-kappaB inhibitors and anti-inflammatory targets based on endothelial cell culture and network pharmacology. *J. Ethnopharmacol.* 174 270–276. 10.1016/j.jep.2015.08.026 26319960

[B18] Kim J. L. Kang S. W. Kang M. K. Gong J. H. Lee E. S. Han S. J. (2012). “Osteoblastogenesis and osteoprotection enhanced by flavonolignan silibinin in osteoblasts and osteoclasts. *J. Cell. Biochem.* 113 247–259. 10.1002/jcb.23351 21898547

[B19] Lane R. K. Hilsabeck T. Rea S. L. (2015). “The role of mitochondrial dysfunction in age-related diseases. *Biochim. Biophys. Acta* 1847 1387–1400. 10.1016/j.bbabio.2015.05.021 26050974 PMC10481969

[B20] Li H. Xie Y. H. Yang Q. Wang S. W. Zhang B. L. Wang J. B. (2012). Cardioprotective effect of paeonol and danshensu combination on isoproterenol-induced myocardial injury in rats. *PLoS ONE* 7:e48872 10.1371/journal.pone.0048872 PMC3490947 23139821

[B21] Li J. He W. Liao B. Yang J. (2015). FFA-ROS-P53-mediated mitochondrial apoptosis contributes to reduction of osteoblastogenesis and bone mass in type 2 diabetes mellitus. *Sci. Rep.* 5:12724 10.1038/srep12724 PMC4521203 26226833

[B22] Li L. Rossoni G. Sparatore A. Lee L. C. Del Soldato P. Moore P. K. (2007). Anti-inflammatory and gastrointestinal effects of a novel diclofenac derivative. *Free Radic. Biol. Med.* 42 706–719. 10.1016/j.freeradbiomed.2006.12.011 17291994

[B23] Li Z. Dong X. Liu H. Chen X. Shi H. Fan Y. (2013). Astaxanthin protects ARPE-19 cells from oxidative stress via upregulation of Nrf2-regulated phase II enzymes through activation of PI3K/Akt. *Mol. Vis.* 19 1656–1666. 23901249 PMC3725964

[B24] Liu H. Bian W. Liu S. Huang K. (2012). Selenium protects bone marrow stromal cells against hydrogen peroxide-induced inhibition of osteoblastic differentiation by suppressing oxidative stress and ERK signaling pathway. *Biol. Trace Elem. Res.* 150 441–450. 10.1007/s12011-012-9488-4 22890880

[B25] Liu Y. H. Lu M. Xie Z. Z. Hua F. Xie L. Gao J. H. (2014). Hydrogen sulfide prevents heart failure development via inhibition of renin release from mast cells in isoproterenol-treated rats. *Antioxid. Redox. Signal.* 20 759–769. 10.1089/ars.2012.4888 23581627

[B26] Lu H. Tian A. Wu J. Yang C. Xing R. Jia P. (2014). Danshensu inhibits beta-adrenergic receptors-mediated cardiac fibrosis by ROS/p38 MAPK axis. *Biol. Pharm. Bull.* 37 961–967. 10.1248/bpb.b13-00921 24882408

[B27] Lu M. Zhao F. F. Tang J. J. Su C. J. Fan Y. Ding J. H. (2012). The neuroprotection of hydrogen sulfide against MPTP-induced dopaminergic neuron degeneration involves uncoupling protein 2 rather than ATP-sensitive potassium channels. *Antioxid. Redox. Signal.* 17 849–859. 10.1089/ars.2011.4507 22360462 PMC3392622

[B28] Maggio D. Barabani M. Pierandrei M. Polidori M. C. Catani M. Mecocci P. (2003). Marked decrease in plasma antioxidants in aged osteoporotic women: results of a cross-sectional study. *J. Clin. Endocrinol. Metab.* 88 1523–1527. 10.1210/jc.2002-021496 12679433

[B29] Manolagas S. C. (2010). From estrogen-centric to aging and oxidative stress: a revised perspective of the pathogenesis of osteoporosis. *Endocr. Rev.* 31 266–300. 10.1210/er.2009-0024 20051526 PMC3365845

[B30] Marshall C. J. (1995). “Specificity of receptor tyrosine kinase signaling: transient versus sustained extracellular signal-regulated kinase activation. *Cell* 80 179–185. 10.1016/0092-8674(95)90401-8 7834738

[B31] Muthusami S. Ramachandran I. Muthusamy B. Vasudevan G. Prabhu V. Subramaniam V. (2005). Ovariectomy induces oxidative stress and impairs bone antioxidant system in adult rats. *Clin. Chim. Acta* 360 81–86. 10.1016/j.cccn.2005.04.014 15970279

[B32] Pan T. T. Feng Z. N. Lee S. W. Moore P. K. Bian J. S. (2006). Endogenous hydrogen sulfide contributes to the cardioprotection by metabolic inhibition preconditioning in the rat ventricular myocytes. *J. Mol. Cell. Cardiol.* 40 119–130. 10.1016/j.yjmcc.2005.10.003 16325198

[B33] Pan T. T. Neo K. L. Hu L. F. Yong Q. C. Bian J. S. (2008). H2S preconditioning-induced PKC activation regulates intracellular calcium handling in rat cardiomyocytes. *Am. J. Physiol. Cell Physiol.* 294 C169–C177. 10.1152/ajpcell.00282.2007 17989210

[B34] Park B. G. Yoo C. I. Kim H. T. Kwon C. H. Kim Y. K. (2005). Role of mitogen-activated protein kinases in hydrogen peroxide-induced cell death in osteoblastic cells. *Toxicology* 215 115–125. 10.1016/j.tox.2005.07.003 16125295

[B35] Satoh T. McKercher S. R. Lipton S. A. (2013). Nrf2/ARE-mediated antioxidant actions of pro-electrophilic drugs. *Free Radic. Biol. Med.* 65 645–657. 10.1016/j.freeradbiomed.2013.07.022 23892355 PMC3859717

[B36] She C. Zhu L. Q. Zhen Y. F. Wang X. D. Dong Q. R. (2014). Activation of AMPK protects against hydrogen peroxide-induced osteoblast apoptosis through autophagy induction and NADPH maintenance: new implications for osteonecrosis treatment? *Cell. Signal.* 26 1–8. 10.1016/j.cellsig.2013.08.046 24080159

[B37] Sikka S. C. (1996). Oxidative stress and role of antioxidants in normal and abnormal sperm function. *Front. Biosci.* 1:e78–e86 10.2741/A146 9159248

[B38] Sivarajah A. McDonald M. C. Thiemermann C. (2006). The production of hydrogen sulfide limits myocardial ischemia and reperfusion injury and contributes to the cardioprotective effects of preconditioning with endotoxin, but not ischemia in the rat. *Shock* 26 154–161. 10.1097/01.shk.0000225722.56681.64 16878023

[B39] Sparatore A. Perrino E. Tazzari V. Giustarini D. Rossi R. Rossoni G. (2009). Pharmacological profile of a novel H(2)S-releasing aspirin. *Free Radic. Biol. Med.* 46 586–592. 10.1016/j.freeradbiomed.2008.11.013 19100325

[B40] Tay A. S. Hu L. F. Lu M. Wong P. T. Bian J. S. (2010). Hydrogen sulfide protects neurons against hypoxic injury via stimulation of ATP-sensitive potassium channel/protein kinase C/extracellular signal-regulated kinase/heat shock protein 90 pathway. *Neuroscience* 167 277–286. 10.1016/j.neuroscience.2010.02.006 20149843

[B41] Tiong C. X. Lu M. Bian J. S. (2010). “Protective effect of hydrogen sulphide against 6-OHDA-induced cell injury in SH-SY5Y cells involves PKC/PI3K/Akt pathway. *Br. J. Pharmacol.* 161 467–480. 10.1111/j.1476-5381.2010.00887.x 20735429 PMC2989596

[B42] Wu H. Krishnakumar S. Yu J. Liang D. Qi H. Lee Z. W. (2014). Highly selective and sensitive near-infrared-fluorescent probes for the detection of cellular hydrogen sulfide and the imaging of H2S in mice. *Chem. Asian J.* 9 3604–3611. 10.1002/asia.201402860 25263845

[B43] Xi J. C. Zang H. Y. Guo L. X. Xue H. B. Liu X. D. Bai Y. B. (2015). The PI3K/AKT cell signaling pathway is involved in regulation of osteoporosis. *J. Recept. Signal Transduct. Res.* 35 640–645. 10.3109/10799893.2015.1041647 26390889

[B44] Xie L. Tiong C. X. Bian J. S. (2012). Hydrogen sulfide protects SH-SY5Y cells against 6-hydroxydopamine-induced endoplasmic reticulum stress. *Am. J. Physiol. Cell Physiol.* 303 C81–C91. 10.1152/ajpcell.00281.2011 22555844

[B45] Xu Z. S. Wang X. Y. Xiao D. M. Hu L. F. Lu M. Wu Z. Y. (2011). “Hydrogen sulfide protects MC3T3-E1 osteoblastic cells against H2O2-induced oxidative damage-implications for the treatment of osteoporosis. *Free Radic. Biol. Med.* 50 1314–1323. 10.1016/j.freeradbiomed.2011.02.016 21354302

[B46] Yang Y. Su Y. Wang D. Chen Y. Wu T. Li G. (2013). Tanshinol attenuates the deleterious effects of oxidative stress on osteoblastic differentiation via Wnt/FoxO3a signaling. *Oxid. Med. Cell. Longev.* 2013:351895 10.1155/2013/351895 PMC3893867 24489983

[B47] Yong Q. C. Lee S. W. Foo C. S. Neo K. L. Chen X. Bian J. S. (2008a). Endogenous hydrogen sulphide mediates the cardioprotection induced by ischemic postconditioning. *Am. J. Physiol. Heart Circ. Physiol.* 295 H1330–H1340. 10.1152/ajpheart.00244.2008 18660450

[B48] Yong Q. C. Pan T. T. Hu L. F. Bian J. S. (2008b). Negative regulation of beta-adrenergic function by hydrogen sulphide in the rat hearts. *J. Mol. Cell Cardiol.* 44 701–710. 10.1016/j.yjmcc.2008.01.007 18329040

[B49] Yu C. Qi D. Lian W. Li Q. Z. Li H. J. Fan H. Y. (2014). Effects of danshensu on platelet aggregation and thrombosis: in vivo arteriovenous shunt and venous thrombosis models in rats. *PLoS ONE* 9:e110124 10.1371/journal.pone.0110124 PMC4222847 25375124

[B50] Yu F. Li P. Song P. Wang B. Zhao J. Han K. (2012). An ICT-based strategy to a colorimetric and ratiometric fluorescence probe for hydrogen sulfide in living cells. *Chem. Commun. (Camb)* 48 2852–2854. 10.1039/c2cc17658k 22293939

[B51] Zhang Z. Zheng L. Zhao Z. Shi J. Wang X. Huang J. (2014). “Grape seed proanthocyanidins inhibit H2O2-induced osteoblastic MC3T3-E1 cell apoptosis via ameliorating H2O2-induced mitochondrial dysfunction. *J. Toxicol. Sci.* 39 803–813. 10.2131/jts.39.803 25242411

